# Improving equity in malaria treatment: Relationship of socio-economic status with health seeking as well as with perceptions of ease of using the services of different providers for the treatment of malaria in Nigeria

**DOI:** 10.1186/1475-2875-7-5

**Published:** 2008-01-08

**Authors:** Obinna Onwujekwe, Benjamin Uzochukwu, Soludo Eze, Eric Obikeze, Chijioke Okoli, Ogbonnia Ochonma

**Affiliations:** 1 Department of Health Administration and Management, College of Medicine, University of Nigeria, Enugu, Nigeria; 2 Health Policy Research Group, Department of Pharmacology and Therapeutics, College of Medicine, University of Nigeria, Enugu, Nigeria; 3 Department of Community Medicine, College of Medicine, University of Nigeria, Enugu, Nigeria

## Abstract

**Background:**

Equitable improvement of treatment-seeking for malaria will depend partly on how different socio-economic groups perceive the ease of accessing and utilizing malaria treatment services from different healthcare providers. Hence, it was important to investigate the link between socioeconomic status (SES) with differences in perceptions of ease of accessing and receiving treatment as well as with actual health seeking for treatment of malaria from different providers.

**Methods:**

Structured questionnaires were used to collect data from 1,351 health providers in four malaria-endemic communities in Enugu state, southeast Nigeria. Data was collected on the peoples' perceptions of ease of accessibility and utilization of different providers of malaria treatment using a pre-tested questionnaire. A SES index was used to examine inequities in perceptions and health seeking.

**Results:**

Patent medicine dealers (vendors) were the most perceived easily accessible providers, followed by private hospitals/clinics in two communities with full complement of healthcare providers: public hospital in the community with such a health provider and traditional healers in a community that is devoid of public healthcare facilities. There were inequities in perception of accessibility and use of different providers. There were also inequity in treatment-seeking for malaria and the poor spend proportionally more to treat the disease.

**Conclusion:**

Inequities exist in how different SES groups perceive the levels of ease of accessibility and utilization of different providers for malaria treatment. The differentials in perceptions of ease of access and use as well as health seeking for different malaria treatment providers among SES groups could be decreased by reducing barriers such as the cost of treatment by making health services accessible, available and at reduced cost for all groups.

## Background

Equitable improvement of treatment-seeking for malaria will depend partly on how different socio-economic groups perceive the ease of accessing and utilizing malaria treatment services from different healthcare providers. Malaria is a major problem in Nigeria and several global and regional targets such as those under the millennium development goals (MDG) and Roll Back Malaria (RBM) have been set in order to encourage malaria-endemic communities to control the disease. Treatment of malaria poses a serious challenge in Nigeria where the disease is a major cause of morbidity and mortality [1]. Understanding socio-economic status (SES) differences in perception of ease of access, perception of ease of utilization as well as health seeking are important for improving the current situation of inequitable provision and utilization of malaria treatment services [1].

Hence, knowledge about the relative perceived ease that different SES groups have for accessing and utilizing malaria treatment services from different providers as well as influence of SES on health seeking will provide an evidence-based decision making for developing frameworks for policy and programmatic interventions for improving equitable treatment-seeking for malaria leading to consumers seeking prompt and appropriate treatment. Some authors have raised the issue that poorer populations may be at risk of contracting malaria, as it seems that they have less access to effective means of treatment once infected [2].

The harsh economic situation of Nigeria and in many sub-Saharan African countries has led to many households especially those from poor SES not seeking care in formal health facilities or delaying the time to seek for formal care for malaria, thus contributing to the high mortality and morbidity rates from the disease. Nigerians face a range of treatment options when ill. These include public sector health facilities and a range of formal and informal private sector health facilities [3]. The seemingly unending economic difficulties have brought about serious increase on informal private sector in treatment provision. This sector which is likely to offer very low quality treatment is also likely to be a more important source of malaria treatment for the poor [4].

There is paucity of knowledge about how socio-economic status (SES) explains the perceptions of ease of accessibility of different healthcare providers determine health-seeking behaviour and utilization of malaria treatment services by different SES groups. Also, there is little information about the link of SES with health seeking for the treatment of malaria in Nigeria. Such evidence is needed to develop strategies for equitable access and treatment of malaria. The problem of accessibility is linked to the cost of obtaining those services, either for the monetary price charged for consultation and drugs, or for the time required to get to the location of the health facility and the resultant effect is inequities among the different socio-economic groups' access to health facilities [5]. Therefore, there is bound to be socio-economic differences in health services [6]. Rich SES groups are likely to have greater availability of, and better access to health services.

In developing countries, it is likely that the limitations set by lack of resources to gain access to good quality health care services are important reasons that poorer households do not readily access healthcare services [7]. A study of the magnitude and nature of socioeconomic differences in the utilization of outpatient health care services showed that utilization among those who report an illness has a clear trend in favour of the wealthier [7]. Only 25% of adults who reported being sick consulted a formal health facility, while for the richest quintile the figure reached 48% [7-9]. Also, it was shown that inequity exists between the rural/urban in their access and utilization of health facilities in Nigeria as more private and general hospitals are located in urban areas than in the rural areas [10]. Other studies in Nigeria and in other parts of sub-Saharan Africa provide some evidence of inequity in access and utilization of malaria treatment services.

Peoples' perception of the ease of accessing the various providers of malaria treatment can potentially determine their health-seeking behaviour. There are indications that delays in receiving care at public hospitals, lackadaisical attitude of the health personnel, distance, etc. have made a shift in the utilization of public services thereby increasing the use of other treatment sources such as private health facilities, drug vendors, and traditional healers [11,12]. Some authors have equally attributed the high patronage of patent medicine dealers to the absence of any public or private facility in within the community [13]. Factors that influence which treatment sources people seek may depend, among other factors on proximity of facility, accessibility, and socioeconomic status of the consumers [14]. This affects both individual and household decision making as to which type of facility to visit, public or private [15].

Inequity in provision of treatment has remained the major reason why alternative, and often times unorthodox and ineffective medicine are sort by consumers. It is important to ensure that ways of improving malaria treatment are equitably considered to accommodate all economic groups. The key issues in ensuring equity for the treatment of malaria would include developing mechanisms that ensure that services are responsive to users and avoiding of polarization of services between rich and poor [16].

There is the need for information on SES differentials in perception of ease of accessing and utilizing the various health care providers as well as SES differences in actual health seeking for developing how policy makers could address inequity in accessibility and utilization of health care services. There is also need for information about the potential level of depletion of household income of different SES groups by malaria, as a pointer to the level of potential catastrophic costs of the disease [17].

The paper hence determined the level of SES differentials in perception of accessibility and utilization of different providers of malaria treatment and how the results can be used to improve malaria treatment services among the various socioeconomic groups. The paper also examines the level of socio-economic inequities in malaria treatment as well as the differences in cost of malaria treatment among the socio-economic groups.

## Methods

### Study area

The study areas were four malaria-endemic communities (towns) in Enugu State, Southeast Nigeria, namely Udi and Nachi in Udi Local government area (LGA), and Inyi and Oji in Oji-River LGA. Udi and Oji are the LGA headquarters, while Nachi and Inyi are not. Each town has a population of at least 20,000 people, while majority of the residents are either subsistence farmers or small time traders. Each town is comprised of at least seven component villages and is an autonomous unit headed by a traditional ruler. Udi and Oji have a minimum of a government owned general hospital and a primary health care centre, together with private hospitals/clinics to complement the public providers. There is a comprehensive health centre and a primary health centre in Inyi, while Nachi is devoid of the presence of any public healthcare provider. Patent medicine stores, itinerant drug providers and herbalists can be found in these towns. *Plasmodium falciparum *causes more than 90% of all malaria cases in the study area [18].

### Contextual framework

The framework of the study is based on the premise that in order to finally consume malaria treatment services, three stages are involved. Stage 1 is concerned with the patient deciding on where to receive treatment in terms of geographic access, which is usually linked to geographic proximity of the healthcare provider. It also depends on the severity of illness. In stage 2, which occurs after the patient has visited the provider the next consideration is how easy it is to consult/see a healthcare provider in the facility visited. Stage 3 deals with issues of actually receiving definitive treatment in terms of collection of drugs. Hence, the manner that different SES groups perceive the three stages has a direct bearing of their level of accessibility of the different providers of malaria treatment services. Based on the contextual framework, operational definitions for the three stages as applied in the study are: (1) Near (geographic proximity): It refers to geographic access; (2) Ease of accessing or attending: It refers to the processes whereby patients get to a health facility, get registered and allowed to see a provider that would diagnose and prescribe treatment; and (3) Ease of receiving treatment: It refers to processes of collection of drugs.

### Study design

A cross-sectional design was used and data was collected using a household survey. In each community four villages were selected by simple random sampling from a sample frame of a list of the villages. A listing of households in each selected village was undertaken to produce the sampling frame. Using the sampling frame, 370 households out of approximately 1,100 households per community were selected from the villages within each community using simple random sampling, with each village contributing equal numbers of households. In each selected household, one woman (primary care giver) or in her absence, male head of household was interviewed using a pre-tested questionnaire. The sample size for the study was a maximum of 300 respondents in each community which was based on an average malaria incidence rate of 10 – 15% in Enugu state [18], 95% confidence level, and 80% power. However, in order to control for refusals and incomplete questionnaires, 370 respondents were selected and approached for interview in each community.

The questionnaire was divided into different sections. The first section was used to collect socio-demographic data about the respondent and his/her household. The second section was used to collect data on actual malaria treatment-seeking behaviour, using one month recall period. In examining treatment-seeking behaviour, the questionnaire explored: How the respondents knew that they had malaria; no of days they were sick with malaria; whether they sought treatment; number of days that elapsed between the time they noticed that they were ill and the time they sought treatment; where they sought treatment and the reasons for doing so; amount of money that they paid to receive treatment; and cost of transportation to receive the treatment. The third section collected data about respondents' perceptions of ease of accessing and utilizing the services of different providers using a series of three questions to ask about the different attributes. The respondents were first presented with the wide choice of the different malaria treatment providers and were first asked how near to them the providers were. They state either yes or no to each provider and were allowed multiple answers. Then, in a similar manner, the respondents were asked how easy it was for them to actually attend the different providers for the treatment of malaria and lastly how easy it was for them to receive malaria treatment services from the providers. The last section of the questionnaire was used to collect data on household asset holdings as well as food expenditure.

### Data analysis

Tabulations were used to analyse the quantitative data. The cost of treatment was computed as treatment cost plus transportation cost. Principal components analysis (PCA) was used to generate an asset-based household socio-economic status (SES) index [14,19] that was used to investigate the equity implications of the findings. Information on ownership of a radio, bicycle, motorcycle, motorcar, refrigerator, together with the weekly household cost of food was used to generate the index.

The SES index was used to divide the households into SES terciles, which were then used to determine the equity implications of some of the key variables. The three SES groups were: the highest SES group (Q3) or least poor; middle SES group (Q2) or average; and lowest SES group (Q1) or most poor. Three SES groups were used instead of the more widely used five groups (quintiles) and four groups (quartiles) because the socio-economic class differences in the rural communities are narrow because of similar income generation activities at that level. Hence, it is more realistic to use two or three SES groups to differentiate the households rather than quintiles or quartiles. Chi-square analysis for trend was used to determine the statistical significance of the differentiation of the dependent variables into SES terciles. The measure of inequity was the concentration index [20,21]. The concentration index varies from -1 and +1 and a negative sign shows that the variable of interest is higher among the poorest and if positive, it means that it is more among the richest (or least poor).

## Results

### Socio-economic and demographic characteristics of the respondents and their households

The number of questionnaires that were completed and acceptable for data analysis in the four groups of respondents was 356 in Inyi, 326 in Udi, 346 in Oji-river (Oji), 323 in Nachi (Table 1). Most of the respondents were females and they were either the wives or representatives of the household heads. Majority of the respondents from the local government headquarters (Oji and Udi) had some level of formal education but majority of respondents were not educated in Nachi while in Inyi it was 50%. Most of the households had approximately four residents, with the highest number of residents per household were from Inyi. The household food costs were highest in Inyi. Radio sets were the commonest movable asset owned by households while motorcar was the least common asset by households. Most households from Oji owned refrigerators.

**Table 1:** Socio-economic and demographic characteristics of the respondents and their households

	Inyi	Udi	Oji	Nachi
	N = 356	N = 326	N = 346	N = 323
	n (%)	n (%)	n (%)	n (%)
Status (spouse/rep)	309 (86.8)	267 (81.9)	310 (89.6)	281 (87.0)
Attended School	178 (50.0)	210 (64.4)	322 (93.1)	126 (39.0)
School years: Mean (SD)	4.50 (5.37)	5.54 (5.09)	9.81 (5.18)	2.97 (4.38)
Married	313 (87.9)	285 (87.4)	339 (98.0)	311 (96.3)
People in house: Mean (SD)	6.30 (3.45)	4.48 (5.55)	5.71 (2.10)	3.81 (2.14)
MALE respondent	23 (6.5)	3 (0.9)	18 (5.2)	16 (5.0)
AGE: Mean (SD)	42.44 (14.45)	43.08 (17.30)	39.00 (11.21)	51.68 (14.49)
Weekly food cost: Mean (SD)	2071.9 (2313.4)	1800.7 (1912.6)	2014.5 (1618.7)	983.5 (1872.4)
Own radio set	292 (82.0)	290 (89.0)	330 (95.4)	279 (86.4)
Own bicycle	255 (71.6)	53 (16.3)	41 (11.8)	136 (42.1)
Own motorcycle	91 (25.6)	40 (12.3)	86 (24.9)	17 (5.3)
Own motorcar	27 (7.6)	41 (12.6)	61 (17.6)	5 (1.5)
Own refrigerator	17 (4.8)	102 (31.3)	252 (72.8)	23 (7.1)

### Experiences with malaria

While a slight majority (56.5%) of the respondents in Inyi had malaria within a month to the date of the interview, minority of the respondents in the other three communities had malaria (Table 2). Most of the people that had malaria sought one form of treatment or the other for the illness, although the lowest proportion that sought treatment was found in Oji community (77.14%). The longest delays before seeking treatment were found in Nachi and the shortest delays in Oji. The longest duration of illness was found in Udi and Inyi at approximately nine days respectively.

**Table 2:** Experiences with malaria

	Inyi	Udi	Oji	Nachi
Respondents that had malaria: n (%)	201 (56.5)	94 (28.8)	105 (30.3)	116 (35.9)
Respondents that sought treatment: n (%)	190/201 (94.52%)	93/94 (98.94%)	81/105 (77.14%)	114/116 (98.28%)
Days elapsed before seeking treatment	1.98 (2.58)	1.62 (1.52)	1.50 (1.65)	2.54 (1.30)
Mean (SD)				
Days malaria lasted: Mean (SD)	8.97(11.18)	9.19(10.45)	4.52 (3.10)	7.89 (4.45)

### Perceptions of ease of accessing and utilizing malaria treatment services

Overall, the patent medicine dealers (PMDs) were the providers that were perceived to be geographically most accessible to the people in the communities, with the exception of Oji where it was public hospital (Table 3). The next nearest set of providers to the people were private hospital in Inyi and public hospital in Udi, while it was traditional healers in Nachi and patent medicine dealers in Oji. The public hospitals were not near to people from Nachi and the community-health workers (CHWs) were not near to the people at all they were non-existent in the study areas. The patent medicine dealers were the providers that people perceived most easily accessed for services for the treatment of malaria in all the groups, except for Oji, where it was public hospital. Table 3 also shows that the majority of respondents generally found that it was easy to receive treatment from patent medicine dealers, except in Nachi, where the majority of the respondents found it easy to receive treatment from herbalists.

**Table 3:** Perceptions of: geographic proximity, ease of accessibility of services and ease of receiving treatment from different healthcare providers

	Inyi	Udi	Oji	Nachi
	n %	n %	n %	n %
**Perceptions of near (geographic proximity)**				

Traditional healer	153 43.0	114 35.0	79 22.8	307 95.0
Private hospital	266 74.7	256 78.5	179 51.7	50 15.5
Public hospital	76 21.3	282 86.5	235 67.9	4 1.2
Patent medicine dealer	272 76.4	318 97.5	230 66.5	315 97.5
Community-health workers (CHW)	167 46.9	69 21.2	49 14.2	52 16.1
Health Center	168 47.2	210 64.4	59 17.1	178 55.1

**Perceptions of ease of accessing the services**				

Traditional healer	160 44.9	100 30.7	68 19.7	305 94.4
Private hospital/clinic	232 65.2	185 56.7	186 53.8	46 14.2
General hospital/comprehensive health centre	94 26.4	200 61.3	238 68.8	3 0.9
Patent medicine dealers	264 74.2	292 89.6	231 66.8	307 95.0
Community-health worker	147 41.3	23 7.1	42 12.1	54 16.7
Health Center	131 36.8	122 37.4	68 19.7	160 49.5

**Perceptions of ease of receiving treatment**				

Traditional healer	147 41.3	103 31.6	67 19.4	311 96.3
Private hospital	215 60.4	203 62.3	189 54.6	81 25.1
Public hospital	99 27.8	175 53.7	237 68.5	20 6.2
Patent medicine dealers	259 72.8	295 90.5	226 65.3	297 92.0
Community-health workers	138 38.8	25 7.7	44 12.7	79 24.5
Health Center	137 38.5	116 35.6	59 17.1	100 31.0

There were some SES differences in perceptions of proximity of the different healthcare providers were to the people in some of the study areas (Table 4). From the statistically significantly differences, the results show that apart from healthcare centre in Nachi, the most poor SES groups did not perceive all other healthcare providers to be near to them when compared to the average least poor SES groups. As was seen in the case of geographic access, there were statistically significant SES differences in perceptions of ease of accessing services, with average and least poor SES perceiving more ease of access to the providers compared to most poor SES (Table 5). In the instances where the results were statistically significant (in Inyi and Oji), the most poor respondents found it most difficult to access the services of the various healthcare facilities/providers. These were in cases of visits to herbalists in Inyi and Oji, private hospitals, public hospitals and patent medicine dealers in Oji, and community health workers and health center in Inyi.

**Table 4:** SES differences in perceptions of geographic proximity of the health care providers

	Inyi	Udi	Oji	Nachi
	n (%)	n (%)	n (%)	n (%)
Traditional healer				
Q1: most poor	45 (29)	37 (32)	13 (16)	107 (35)
Q2:average	58 (38)	41 (36)	35 (44)	101 (33)
Q3: least poor	50 (33)	36 (32)	31 (39)	99 (32)
Chi square (p-value)	2.9 (0.2)	0.5 (0.8)	13.5 (0.001)	3.4 (0.2)
Concentration index	0.03	0.00	0.11	-0.01

Private hospital				
Q1: most poor	77 (29)	86 (34)	31 (17)	7 (14)
Q2:average	98 (37)	83 (32)	80 (45)	21 (42)
Q3: least poor	91 (34)	87 (34)	68 (38)	22 (44)
Chi square (p-value)	10.3 (0.01)	0.6 (0.7)	44.9 (0.0001)	10.4 (0.01)
Concentration index	0.03	0.02	0.15	0.21

Public hospital				
Q1: most poor	25 (33)	91 (32)	53 (23)	0 (0)
Q2:average	22 (29)	92 (33)	95 (40)	3 (75)
Q3: least poor	29 (38)	99 (35)	87 (37)	1 (25)
Chi square (p-value)	1.3 (0.5)	3.7 (0.2)	39.8 (.0001)	3.5 (0.2)
Concentration index	0.03	0.02	0.10	

Patent medicine dealers				
Q1: most poor	80 (29)	105 (33)	59 (25)	104 (33)
Q2:average	93 (34)	108 (34)	84 (37)	106 (34)
Q3: least poor	99 (36)	105 (33)	87 (38)	105 (33)
Chi square (p-value)	9.4 (0.01)	1.8 (0.4)	20.2 (0.0001)	3.2 (0.2)
Concentration index	0.06	0.00	0.10	0.01

Community-health workers				
Q1: most poor	47 (28)	18 (26)	9 (18)	9 (17)
Q2:average	65 (39)	22 (32)	17 (35)	22 (42)
Q3: least poor	55 (33)	29 (42)	23 (47)	21 (40)
Chi square (p-value)	5.5 (0.06)	3.6 (0.2)	7.7 (0.022)	7.5 (0.02)
Concentration index	0.03	0.11	0.21	0.14

Health centre				
Q1: most poor	45 (27)	68 (32)	24 (41)	71 (40)
Q2:average	63 (37)	72 (34)	19 (32)	54 (30)
Q3: least poor	60 (36)	70 (33)	16 (27)	53 (30)
Chi square (p-value)	6.4 (0.04)	0.3 (0.8)	1.8 (0.41)	6.7 (0.04)
Concentration index	0.00	0.02	-0.08	-0.15

**Table 5:** SES differences in perceptions of ease of accessing the services of healthcare providers

	Inyi	Udi	Oji	Nachi
	n (%)	n (%)	n (%)	n (%)
Traditional healer				
Q1: most poor	43 (27)	32 (32)	14 (20)	104 (34)
Q2:average	64 (40)	41 (41)	27 (40)	103 (34)
Q3: least poor	53 (33)	27 (27)	27 (40)	98 (32)
Chi square (p-value)	7.5 (0.02)	4.2 (0.1)	6.4 (.04)	1.2 (0.6)
Concentration index	0.04	-0.03	0.14	-0.01

Private hospital				
Q1: most poor	69 (30)	68 (37)	34 (34)	10 (22)
Q2:average	82 (35)	61 (33)	83 (45)	18 (39)
Q3: least poor	81 (35)	56 (30)	69 (37)	18 (39)
Chi square (p-value)	4.1 (0.1)	2.5 (0.3)	43.7 (.0001)	3.5 (0.2)
Concentration index	0.03	-0.05	0.14	0.12

Public hospital				
Q1: most poor	30 (32)	59 (30)	53 (22)	1 (33)
Q2:average	35 (37)	72 (36)	96 (40)	1 (33)
Q3: least poor	29 (31)	69 (34)	89 (37)	1 (33)
Chi square (p-value)	0.8 (0.7)	3.7 (0.2)	43.5 (.0001)	0.0 (0.9)
Concentration index	0.00	0.01	0.12	na

Patent medicine dealers				
Q1: most poor	80 (30)	100 (34)	59 (26)	104 (34)
Q2:average	92 (35)	97 (33)	84 (36)	99 (32)
Q3: least poor	92 (35)	95 (33)	88 (38)	104 (34)
Chi square (p-value)	4.5 (0.1)	0.9 (0.6)	21.3 (.0001)	4.8 (0.09)
Concentration index	0.03	-0.01	0.09	0.00

Community-health workers				
Q1: most poor	40 (27)	8 (35)	9 (21)	13 (24)
Q2:average	56 (38)	6 (26)	18 (43)	21 (39)
Q3: least poor	51 (35)	9 (39)	15 (36)	20 (37)
Chi square (p-value)	4.7 (0.09)	0.7 (0.7)	3.3 (.19)	2.7 (0.3)
Concentration index	0.05	-0.01	0.12	0.09

Health centre				
Q1: most poor	34 (26)	44 (36)	26 (38)	60 (38)
Q2:average	44 (34)	42 (34)	21 (31)	50 (31)
Q3: least poor	53 (40)	36 (30)	21 (31)	50 (31)
Chi square (p-value)	6.8 (0.03)	1.2 (0.5)	.88 (.65)	2.0 (0.4)
Concentration index	0.01	-0.04	-0.13	-0.04

There were evidences of socioeconomic inequity in perceived ease of receiving treatment for malaria from the various healthcare providers (Table 6). As in the case of perceptions of geographic accessibility and ease of receiving treatment, the average and least poor SES groups stated that it was easier for them to receive treatment from healthcare providers compared to most-poor SES group, with the exception of traditional healers and health centers in Nachi. In Inyi the highest proportion of respondents who perceived that it was easy to receive treatment from public hospitals belonged to the average SES group (p < 0.05). A similar result was found in the use of herbalists, private hospital, and public hospital in Oji. Statistically significant inequities were found in the use of community health workers in Inyi and use of patent medicine dealers in Oji. In Nachi, the highest proportion of people that found that it was easy to receive malaria treatment services from herbalists and health centre were from the most poor SES followed by the average SES group (p < 0.05).

**Table 6:** SES differences in perceptions of ease of receiving treatment for malaria from providers

	Inyi	Udi	Oji	Nachi
	n (%)	n (%)	n (%)	n (%)
Traditional healer				
Q1: most poor	42 (29)	34 (33)	14 (21)	109 (35)
Q2:average	58 (39)	40 (39)	27 (40)	104 (33)
Q3: least poor	47 (32)	29 (28)	26 (39)	98 (32)
Chi square (p-value)	4.6 (0.1)	2.4 (0.3)	6.0 (.051)	8.6 (0.01)
Concentration index	0.02	-0.03	0.13	-0.10

Private hospital				
Q1: most poor	66 (31)	67 (33)	35 (19)	20 (25)
Q2:average	75 (35)	64 (32)	82 (43)	26 (32)
Q3: least poor	74 (34)	72 (35)	72 (38)	35 (43)
Chi square (p-value)	1.8 (0.4)	1.5 (0.5)	42.7 (.0001)	6.2 (0.04)
Concentration index	0.02	0.01	0.14	0.07

Public hospital				
Q1: most poor	27 (27)	53 (30)	54 (23)	6 (30)
Q2:average	43 (43)	63 (36)	95 (40)	6 (30)
Q3: least poor	29 (29)	59 (34)	88 (37)	8 (40)
Chi square (p-value)	6.3 (0.04)	1.9 (0.4)	39.1 (.0001)	0.5 (0.8)
Concentration index	0.02	0.03	0.10	0.07

Patent medicine dealers				
Q1: most poor	82 (32)	100 (34)	57 (25)	96 (32)
Q2:average	89 (34)	97 (33)	84 (37)	101 (34)
Q3: least poor	88 (34)	98 (33)	85 (38)	100 (34)
Chi square (p-value)	1.3 (0.5)	0.5 (0.8)	20.7 (.0001)	3.4 (0.2)
Concentration index	0.02	-0.01	0.17	0.02

Community-health workers				
Q1: most poor	36 (26)	8 (32)	11 (25)	28 (35)
Q2:average	51 (37)	6 (24)	18 (41)	30 (38)
Q3: least poor	51 (37)	11 (44)	15 (34)	21 (27)
Chi square (p-value)	5.5 (0.07)	1.7 (0.4)	1.8 (0.40)	2.0 (0.4)
Concentration index	0.00	0.08	0.07	-0.04

Health centre				
Q1: most poor	38 (28)	39 (34)	26 (44)	55 (55)
Q2:average	47 (34)	41 (35)	15 (25)	28 (28)
Q3: least poor	52 (38)	36 (31)	18 (31)	17 (17)
Chi square (p-value)	3.8 (0.2)	0.4 (0.8)	4.0 (0.14)	31.7 (0.01)
Concentration index	0.06	-0.02	-0.08	-0.25

### Treatment-seeking

There was generally statistically insignificant differences in incidence of malaria across the three SES groups in three groups, with the only exception been in Udi where the most poor SES had the lowest incidence of malaria (p = 0.05). Self-diagnosis was the procedure that was used by most of the respondents that had malaria to diagnose the illness. Laboratory tests, though the second most common method of diagnosis was not commonly used by the respondents. It is possible that some respondents used more than one procedure to diagnose their illnesses. Home treatment (treatment at home with already existing/stored drugs without recourse to a health provider) was not a common source of first treatment (Table 7). Traditional medicines were very common sources of treatment in Nachi (27.59%) and they were the third most common source of treatment in Inyi (12.94%).

**Table 7:** Treatment that was sought for malaria

	Inyi	Udi	Oji	Nachi
	n (%)	n (%)	n (%)	n (%)
Home treatment	12 (6.3)	9 (9.6)	5 (6.2)	6 (5.2)
Traditional medicine	25 (13.2)	4 (4.3)	8 (9.9)	32 (28.1)
Private hosp/clinic	35 (18.4)	18 (19.4)	13 (16.0)	18 (15.8)
General hospital	16 (8.4)	17 (18.3)	4 (4.9)	3 (2.6)
Patent medicine dealer	92 (48.4)	36 (38.7)	38 (46.9)	51 (44.8)
CHW	3 (1.6)	1 (1.1)	3 (3.7)	0 0
Health Centre	3 (1.6)	1 (1.1)	1 (1.2)	1 (0.9)
Laboratory	1 (0.5)	4 (4.3)	2 (2.5)	2 (1.8)
Others	3 (1.6)	3 (3.2)	7 (8.6)	1 (0.9)

Distance of the healthcare provider to the consumers was a strong determinant of where people first sought treatment for malaria. Readily availability of drugs was the second overall most important reason that people gave for seeking for care from various providers. The quality of services was also an important determinant of where people first sought treatment, though it had the highest proportion of people in only Inyi.

### SES differences in treatment-seeking and cost of treatment

While the most poor SES were most likely to seek treatment in Oji group (p < 0.05), the least poor SES in Udi had the least delay before seeking care for malaria (p = 0.07). There were statistically insignificant differences in the number of days the ill people had malaria. Concentration index shows that the rich had malaria more than the poor except for Oji where more of the respondents are from the poor group. The results also show that the least poor sought for treatment more than the most poor in all the study areas.

There was some evidence of socio-economic differentials with regards to the providers where treatment was first sought, although some of the directions of inequity were not uniform (Table 8). For instance, while the least poor SES respondents used home treatment more than the most poor in Inyi, the reverse was found in Udi (p < 0.05). However, the most poor SES used more of traditional medicines and least of private hospitals and clinics in Udi (p < 0.05). The remaining statistically significant evidences on socio-economic inequity were found in Nachi, where the most poor SES were most likely to use services of patent medicine dealers and in Udi, where the least poor SES were most likely to use the services of laboratories for the treatment of malaria (p < 0.05). The findings also show that there was no socio-economic difference with regards to the number of people that recovered after the first treatment action that was taken. The study indicates that the poor had more treatment of malaria in Udi while reverse is the case in Nachi and Inyi. The result also shows that in Inyi, the poor will go for traditional medicine, private hospital/clinic and patent medicine dealers more than the rich at concentration index of -0.08, -0.04 and -0.05 respectively.

**Table 8:** SES differences in choice of providers for the treatment of malaria

	Inyi	Udi	Oji	Nachi
	n (%)	n (%)	n (%)	n (%)
Home treatment				
Q1: most poor	0 (0)	2 (25.0)	4 (80.0)	2 (33.3)
Q2:average	6 (50.0)	6 (75.0)	0 (0)	2 (33.3)
Q3: least poor	6 (50.0)	0 (0)	1 (20.0)	2 (33.3)
Chi square (p-value)	6.3 (0.04)	6.4 (0.04)	4.6 (0.10)	2.1 (0.7)
Concentration index	0.34	-0.84	-	0.01

Traditional medicines				
Q1: most poor	8 (32.0)	3 (100.0)	6 (75.0)	10 (31.3)
Q2:average	9 (36.0)	0 (0)	1 (12.5)	6 (18.7)
Q3: least poor	8 (32.0)	0 (0)	1 (12.5)	16 (50.0)
Chi square (p-value)	0.05 (0.9)	10.1 (0.01)	5.3 (0.07)	5.1 (0.08)
Concentration index	-0.08	-	-	0.13

Private hospital				
Q1: most poor	14 (44.8)	2 (12.5)	6 (46.1)	3 (16.7)
Q2:average	6 (18.7)	4 (25.0)	5 (38.5)	6 (33.3)
Q3: least poor	12 (37.5)	10(62.5)	2 (15.4)	9 (50.0)
Chi square (p-value)	4.2 (0.1)	4.8 (0.09)	1.05 (0.6)	3.0 (0.2)
Concentration index	-0.04	0.33	-0.19	0.23

Public hospital				
Q1: most poor	2 (14.3)	5 (31.3)	1 (25.0)	1 (33.3)
Q2:average	7 (50.0)	8 (50.0)	2 (50.0)	1 (33.3)
Q3: least poor	5 (35.7)	3 (18.7)	1 (25.0)	1 (33.3)
Chi square (p-value)	2.8 (0.3)	3.1 (0.2)	.39 (.82)	0.01 (0.9)
Concentration index	0.15	0.12	-	-

Patent medicine dealer				
Q1: most poor	33 (36.7)	9 (25.7)	13 (35.1)	21 (41.1)
Q2:average	30 (33.3)	12 (34.3)	11 (29.8)	19 (37.3)
Q3: least poor	27 (30.0)	14 (40.0)	13 (35.1)	11 (21.6)
Chi square (p-value)	1.1 (0.6)	0.4 (0.8)	4.1 (0.39)	7.7 (0.02)
Concentration index	-0.05	0.10	0.01	-0.11

Primary Healthcare (PHC) centre				
Q1: most poor	0 (0)	0 (0)	0 (0)	0 (0)
Q2:average	1 (33.3)	0 (0)	1 (100)	1 (100)
Q3: least poor	2 (66.3)	1 (100)	0 (0)	0 (0)
Chi square (p-value)	2.1 (0.4)	1.6 (0.4)	1.6 (0.5)	2.2 (0.3)
Concentration index	-	-	-	-

The least poor SES generally spent more money to treat malaria, although the finding was only statistically significant in Nachi (p < 0.05) and slightly significant in Inyi (p < 0.10) (Table 9). Similarly, the least poor SES spent more on transportation to treat malaria and the finding was statistically significant in Udi and Nachi (p < 0.05). However, the opposite was found in Oji where the most poor SES actually spent the highest amount of money on transportation (p < 0.05). In Nachi, there was statistically significant SES differentials in total financial cost to treat malaria, with a progressive increase in costs and one moves from the most to the least poor SES (p < 0.05). The time costs to the least poor households were more as seen in Inyi and Nachi.

**Table 9:** Cost of treatment of malaria

	Inyi	Udi	Oji	Nachi
	Mean (SD)	Mean (SD)	Mean (SD)	Mean (SD)
Treatment cost				
Q1: most poor	140.9 (197.6)	389.1 (478.9)	292.7 (377.9)	214.7 (292.9)
Q2:average	138.4 (165.6)	359.2 (592.2)	311.1 (165.5)	420.8 (687.9)
Q3: least poor	194.1 (193.9)	491.4 (612.0)	157.1 (165.5)	619.0 (907.4)
Chi square	5.1	4.3	0.70	6.7
p-value	0.08	0.1	0.70	0.04

Transportation cost				
Q1: most poor	19.7 (66.8)	35.5 (53.1)	49.7 (115.7)	7.9 (22.7)
Q2:average	7.6 (26.5)	28.3 (53.3)	37.4 (75.2)	23.8 (41.5)
Q3: least poor	17.1 (67.3)	50.3 (46.9)	1.4 (7.6)	41.2 (58.5)
Chi square	0.9	7.5	6.3	12.2
p-value	0.6	0.02	0.04	0.00

Total financial cost				
Q1: most poor	307.9 (574.3)	422.3 (526.0)	301.4 (385.2)	232.1 (352.3)
Q2:average	196.0 (334.5)	935.3(3324.5)	277.9 (369.3)	444.6 (719.5)
Q3: least poor	286.5 (452.4)	544.2 (629.8)	158.6 (165.6)	661.0 (950.0)
Chi square	2.6	3.7	0.7	7.7
p-value	0.3	0.2	0.7	0.02

Time costs				
Q1: most poor	903.6 (2560.5)	843.2 (2168.7)	740.5(1048.0)	564.2 (841.1)
Q2:average	1429.1 (2367.6)	430.3 (727.9)	1101.3 (1920.)	656.5 (950.2)
Q3: least poor	2078.3 (5261.6)	1680.6 (6660.6)	922.9 (1381.8)	1467.3 (2110.8)
Chi square	9.6	0.05	0.7	14.5
p-value	0.01	0.9	0.7	0.00

## Discussion

Patent medicine dealers (vendors) were perceived to be the nearest set of providers to the people in the communities, apart from the findings in only one community where it was the public hospital. This is buttressed by the finding that upon recognition of symptom, most of the respondents go to patent medicine dealers for their treatment, and they often make choices on the kind of drug they would be offered. The treatment options chosen were as a result of the fact that the public healthcare facilities were not readily available especially in the rural areas. Similar studies in Nigeria as well as in the rest of sub-Saharan Africa have also shown that patent medicine dealers are the most accessible source of treatment for malaria [13,22]. The results also show that it was in communities without public healthcare facilities that residents hardly had access to such facilities, a clear reflection that those healthcare facilities were not near to such people.

More than half of the respondents used self recognition to know that they had malaria and such improper diagnosis could lead to irrational drug use, more expenditure on drugs and extension of days of illness. Self diagnosis is misleading when it is recognized that there are other illnesses that have similar symptoms as malaria. Hence, caution should be exercised in adducing all the costs of illness to malaria, since the illnesses could have been caused by other clinical conditions that manifest with fever [3]. Whilst, some studies have found that people of low socio-economic status group were most likely to indulge in self-diagnosis, in India, people from high socio-economic group were most likely to engage in self diagnosis [23].

There was evidence of socio-economic status (SES) differentiation in the perceptions of the respondents about the proximity of the healthcare providers to them. The average and least poor SES groups perceived it easier to access the healthcare providers than the most-poor SES group. Some authors stated that although, people of poorer SES may be at a similar risk of contracting malaria, it seems that they have less access to effective means of treatment once infected [3]. Also, in the perceptions of ease of receiving malaria treatment services from various healthcare facilities, there were traces of inequity, which was tilted against the most-poor SES group.

There were also SES differentials in health seeking for the treatment of malaria in all the study communities and the results reveal that the least poor SES group actually generally sought care more frequently than the most-poor SES group when they are ill, although curiously most-poor households in Inyi sought treatment more that the least poor. Overall, the least poor SES group hence have less delay before seeking treatment unlike the most-poor SES group.

The finding (apart from one of the communities) that the total financial cost of treating an episode of malaria was not significantly different across the three SES groups imply that the most-poor paid more in proportion to their income to obtain malaria treatment. However, it could also be argued that the least poor could have lost more income in absolute terms but not necessarily more than the most-poor relative to their income. The high malaria treatment expenditure could lead to catastrophic expenditures and impoverishment [17], especially viewed from the results where the proportion of malaria treatment expenditure to good expenditure in all the communities was more than 10%. The fact that the time costs of malaria to the least poor households when compared to that from the most-poor households was significantly more intuitively reflects the fact that the higher the SES, the more income that would be lost in terms of illness.

A limitation of the study because of its quantitative nature was the inability to explore reasons behind the perceptions of geographic proximity, ease of accessing services and ease of receiving treatment from various providers. Also, the reasons why about 23% of people that reported that they had malaria in Oji did not seek treatment were not explored in this study, but could have provided more insight into health seeking behaviour for malaria. The SES of the people that did not seek treatment will have also provided additional information that would be useful in developing interventions to improve equity in malaria treatment. Also, many other factors such as recognition of illness, decision to seek treatment, decision where to seek treatment, receipt of prescription for antimalarial drugs, correct administration of drugs and adherence. These issues would be explored in similar studies in future if the opportunity avails.

There is the need to address this issue of inequity in accessibility and health seeking for treatment of malaria so as to ensure optimal levels of access to and utilization of appropriate malaria treatment services for all SES group if the MDG of halving the incidence and burden of malaria by 2015 is to be achieved in Nigeria. Appropriate malaria treatment services should be made both easily geographically and financially accessible to all SES groups, especially the most-poor, especially as was found that distance was a strong determinant of where people first sought treatment. The **c**ost of malaria treatment should be minimized to enable all SES groups to have access drugs when ill. Progressive payment based on SES status could be used to ensure that the most poor do not pay as much as other SES groups for malaria. The quality of services that are offered by patent medicine dealers should be improved since they are the most easily accessible providers and are the first point of call for treatment of malaria. However, public health facilities should be made more accessible to the poor SES, in the form of provision of more functional primary healthcare facilities, with ready availability of drugs. User fee exemption or subsidies for anti malarial drugs can be introduced to allow for increase in utilization of treatment facilities.

## Authors' contributions

OO conceived and designed the study. All the authors participated in data collection and analysis. OO wrote the first draft and all the authors revised the drafts until the final draft was produced for publication.
